# Lyophilized human platelet lysate: manufacturing, quality control, and application

**DOI:** 10.3389/fcell.2025.1513444

**Published:** 2025-01-27

**Authors:** Kerstin Wendland, Lea Koblin, Dirk Stobbe, Anna Dahms, Debora Singer, Sander Bekeschus, Jan Wesche, Janosch Schoon, Konstanze Aurich

**Affiliations:** ^1^ Institute of Transfusion Medicine, University Medicine Greifswald, Greifswald, Germany; ^2^ Center for Orthopaedics, Trauma Surgery and Rehabilitation Medicine, University Medicine Greifswald, Greifswald, Germany; ^3^ ZIK Plasmatis, Leibniz Institute for Plasma Science and Technology (INP), Greifswald, Germany; ^4^ Department of Dermatology and Venerology, Rostock University Medical Center, Rostock, Germany

**Keywords:** human platelet lysate, freeze drying, lyophilization, mesenchymal stromal cells, sustainable cell culture, HPL

## Abstract

**Background:**

A significant number of platelet concentrates (PCs) is discarded daily in blood banks due to limited shelf life. Human platelet lysate (HPL), derived from expired PCs, has gained attention as an ethical and sustainable cell culture media supplement in biomedical research and cell therapy production. However, HPL is subject to decisive disadvantages such as batch differences and lack of storage stability. To overcome these limitations and to enhance the applicability of HPL, we developed an HPL manufacturing protocol including a lyophilization process. The aim of this study was to investigate the influence of HPL lyophilization on parameters of quality control, including growth factor concentrations and the culture of human mesenchymal stromal cells (hMSCs).

**Methods:**

We performed a paired comparison of six batches of HPL and lyophilized HPL (L-HPL) regarding the quality parameters pH, total protein, osmolality, sodium, potassium and chloride concentration. Concentrations of 11 growth factors and cytokines were compared between HPL and L-HPL. Additionally, we determined cell yield, proliferation capacity, viability and trilineage differentiation potential of hMSCs following expansion in HPL- and L-HPL-supplemented cell culture media.

**Results:**

Quantification of the quality parameters revealed non-altered pH, osmolality and potassium concentrations and slightly lower total protein, sodium and chloride concentrations of L-HPL compared to HPL. Growth factor and cytokine concentrations did not differ between HPL and L-HPL. Cell yield, division cycles and viability of hMSCs cultured in either HPL- or L-HPL-containing media were comparable. Cells differentiated in medium containing L-HPL showed a slightly higher capacity for osteogenic differentiation, while adipogenic differentiation and chondrogenic differentiation potentials remained unchanged.

**Conclusion:**

We successfully developed a method to produce well-applicable L-HPL. The comparison of L-HPL with HPL did not reveal any relevant differences regarding quality control parameters of routine testing, growth factor concentrations and hMSC functionality, demonstrating the suitability of L-HPL as a cell culture supplement. These results emphasize the potential of L-HPL as a sustainable and ethical alternative to animal-derived serum products in biomedical research and drug development.

## Introduction

Cell culture media and their supplements play a pivotal role in supporting various cell types used in biomedical research, regenerative medicine and pharmaceutical production. Different cell culture media supplements are used to supply the basal media with essential nutrients such as amino acids, growth factors, vitamins, inorganic salts and lipids, thereby supporting and maintaining cell proliferation and metabolism *in vitro*. The three main categories of media supplements comprise animal-derived serum, xeno-free supplements and chemically defined synthetic supplements (Yao and Asayama, 2017). Among the broad range of animal-derived sera, the most widely used product is fetal calf serum (FCS), with an estimated worldwide usage of over 800.000 L annually (Brindley et al., 2012). Despite its still leading role as cell culture media supplement, the use of FCS is being regarded more and more critically, both from an ethical and scientific standpoint. Issues associated with FCS concern its inhumane and scarcely regulated production methods, its varying composition and strong batch-to-batch variations as well as the risk of transferring xenogenic pathogens (Jochems et al., 2002).

Consequently, alternatives to FCS are being sought after, with human platelet lysate (HPL) presenting a promising option that circumvents the above-mentioned problems of FCS (Subbiahanadar Chelladurai et al., 2021). One possibility to produce HPL is the use of expired human platelet concentrates (PCs) that are no longer suitable for transfusion to patients due to their short shelf life. Thus, HPL is a sustainable product manufactured in a well-regulated laboratory environment which eliminates the potential of xenogenic contamination in human cell cultures. The use of pooled PCs from whole blood donation buffy coats of multiple donors ensures consistent composition between different batches and limits donor-dependent variability (Burnouf et al., 2016). Furthermore, several studies have shown that proliferation and expansion of various cell types improves if HPL is used instead of FCS (Canestrari et al., 2019; Oeller et al., 2021), most notably in the field of human mesenchymal stromal cells (hMSCs) (Guiotto et al., 2020). These cells have gained interest in the field of regenerative medicine due to their multipotent capacities and their ability to support tissue homeostasis upon injury (Sagaradze et al., 2020). Human MSCs can be readily isolated from various tissues (Handke et al., 2023; Ghoneim et al., 2020; de Pedro et al., 2023) and their potential for clinical use relies on the cells’ origin and continued *in vitro* expansion while maintaining a defined hMSC phenotype according to ISCT criteria (Dominici et al., 2006) and multilineage differentiation capacity (Zhou and Shi, 2023). Previous work has shown that expansion of bone marrow derived hMSCs in media containing HPL outperforms hMSCs cultured in media containing FCS in terms of cartilage formation *in vitro* (Brachtl et al., 2022) and that those cartilage discs were successful regarding the regeneration of critical size femoral bone defects in pre-clinical application (Hochmann et al., 2023). This clearly demonstrates that the choice of serum supplements plays a crucial role in directing cell functionality in the context of cell-based therapies. To establish the foundation for the applicability of HPL in cell expansion for cell-based therapies, minimal quality control parameters have recently been defined (De Korte et al., 2024). These parameters include microbiological safety screenings, content quantification of total protein and selected growth factors as well as *in vitro* testing of biological activity in terms of cell proliferation doublings.

Lyophilization of protein-containing media supplements enables weight reduction and increase of thermal stability due to the removal of the solvent (Abdul-Fattah et al., 2007), however, establishing suitable lyophilization protocols can be challenging due to limited physical and chemical stability of proteins (Wang, 2000). Initial reports regarding HPL lyophilization protocols and the application of lyophilized HPL (L-HPL) were recently proposed for the first time, aiming to further improve HPL regarding its applicability in regenerative medicine and experimental cell cultures (Notodihardjo et al., 2019; Marino et al., 2023). However, it remains entirely unclear whether lyophilization impacts the quality of HPL. To date, there are no studies comparing the quality of L-HPL, produced under standardized protocols, with that of conventional HPL. We believe that the lyophilization of HPL can elevate its applicability and quality standardization to the next level. Therefore, in this study, we aimed to 1) establish a production protocol for L-HPL that allows for future production considering good manufacturing practice conditions, and 2) to test L-HPL with respect to the aforementioned quality parameters and other important functional markers, including functional parameters of hMSCs, and to compare these with conventional HPL.

## Material and methods

### Manufacturing of pooled platelet concentrates from whole blood donations

For each pooled PC, four ABO-identical buffy coats from whole blood donations were pooled with 250 mL SSP+ plasma replacement solution (Macopharma), depleted from leukocytes through a filtering system (Fresenius Kabi) and then left to rest for 30 min. The pooled PCs were centrifuged at 700 × g at room temperature (RT) for 4 min (Roto Silenta RS630, Hettich GmbH). The automated MacoPress blood separator (Macopharma) was used to separate red blood cells from the pooled PC. The plasma: SSP+ ratio in each pooled PC was 30:70. Shelf life for clinical use of a pooled PC is 96 h at RT with agitation. All donations are tested according to the European blood guideline (2002/98/EC) for viral safety including HIV1/2, Hepatitis B, C, E, West nile virus and *Treponema pallidum*.

### Manufacturing of lyophilized human platelet lysate

**FIGURE 1:**
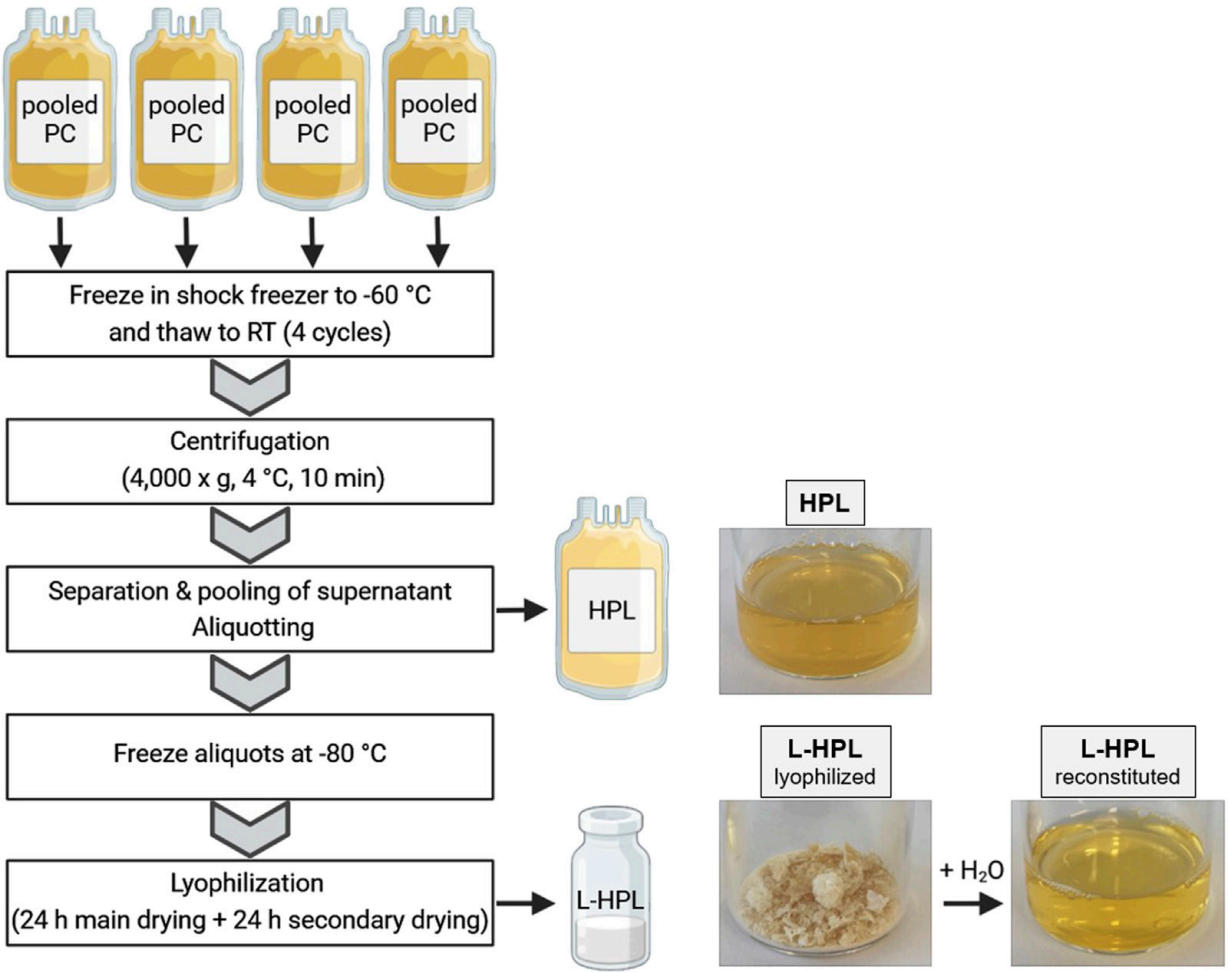
Schematic overview of human platelet lysate (HPL) and lyophilized HPL (L-HPL) manufacturing. Four expired pooled platelet concentrates (PCs) are subjected to four freeze-thaw cycles and then centrifuged to separate the HPL from cell debris. To obtain L-HPL, HPL aliquots are frozen at −80°C and then lyophilized in a two-step freeze-drying process. Photographic images of HPL, L-HPL in lyophilized form and L-HPL in reconstituted form indicate the physical appearance at each manufacturing step. Created in BioRender. Wendland, K. (2025) https://BioRender.com/z42o911.

Immediately after expiring for clinical use, the PCs were shock frozen at −60°C (MBF-21, B-Medical System) and stored at −35°C for an average of 16 days (±11 days) before being used for HPL manufacturing. The use of expired blood products is approved by the local ethic committee of University medicine Greifswald (ethics vote No BB 014/14). Four expired PCs (equals a pool of 16 donors) were used to produce one batch of HPL. Production of HPL was realized in a closed bag system under sterile conditions (Figure 1). To this end, PCs were subjected to four freeze-thaw cycles in a shock freezer (MBF-21; B Medical Systems) to lyse the platelets and then centrifuged at 4,000 × g at 4°C for 10 min (Roto Silenta RS630; Hettich GmbH). Platelet lysis was confirmed with flow cytometry using CD61 and forward/sideward scatter to quantify remaining intact platelets and platelet debris in the lysate (Supplementary Figure S1A). The supernatant was separated from cell debris using a manual plasma extractor (Baxter) and pooled subsequently from all four PCs. After final centrifugation at 4,000 × g at 4°C for 10 min the supernatant was aliquoted in four 125 mL pediatric blood bags (Macopharma) and frozen at −80°C. For lyophilization, the bags were thawed and 25 mL of HPL were transferred to 50 mL conical tubes and horizontally frozen at −80°C. The frozen aliquots were lyophilized using a freeze dryer (Christ Alpha 1–4 LDplus; Christ Gefriertrocknungsanlagen GmbH) with the following settings: primary drying for 24 h at −8°C and 0.1 mbar followed by secondary drying for 24 h at 0°C and 6.1 mbar. After lyophilization, L-HPL was stored at −20°C until reconstitution for subsequent experiments.

### Thawing and reconstitution

A total of six batches of HPL and the corresponding L-HPL were prepared. Both HPL and L-HPL were stored for 3–6 months at −20°C prior to use. HPL was thawed at RT and centrifuged at 4,000 × g for 15 min. The frozen L-HPL was reconstituted with sterile dH_2_O (Merck Millipore) under gentle agitation to reach the initial volume of 25 mL. The HPL supernatants and the reconstituted L-HPL batches were used for the subsequent quality control analyses as well as for the cell culture experiments.

### Isolation and cultivation of mesenchymal stromal cells

Human MSCs were isolated from bone marrow of a 72-year old male patient (donor 1) and a 58-year old male patient (donor 2). The bone marrow samples were harvested during primary hip arthroplasty. This study was approved by the independent ethics committee of the University Medicine Greifswald in accordance with the World Medical Association Declaration of Helsinki (BB 087/21). The patients provided informed written consent. Isolation and cultivation of hMSCs were performed as described previously (Handke et al., 2023; Andrzejewska et al., 2019). In brief, mononuclear cells of the bone marrow (BN-MNCs) were isolated by density gradient centrifugation and hMSCs were isolated via plastic adherence after seeding of BM-MNCs. The cells were cultured at 37°C, 5% CO_2_ and 95% humidity in expansion medium (EM) containing low glucose Dulbecco’s Modified Eagle’s Medium (DMEM, PAN Biotech), 10% (v/v) FCS (Sigma-Aldrich), 100 U/mL penicillin (Gibco), 100 μg/mL streptomycin (Gibco) and 2 mM L-alanyl-L-glutamine (GlutaMAX, Gibco). After 14 days of primary cell culture, the hMSCs were detached with 0.05% trypsin containing 0.02% EDTA (PAN-Biotech) and cryopreserved. After thawing, the cells were cultured in EM containing 10% FCS until confluence of 80% was reached, detached and seeded at a cell density of 2,400 cells/cm^2^ in EM containing either 7% (v/v) HPL or 7% (v/v) reconstituted L-HPL instead of 10% (v/v) FCS. After trypsinization, the cell numbers were counted using the TC20 Automated Cell Counter (Bio-Rad Laboratories) to quantify cell yield.

### Quality control of lyophilized human platelet lysate

#### Quantification of quality control parameters using routine testing of blood products

Quality control parameters that were covered by routine testing during standardized production of blood products in our department were applied for the quality control of HPL and L-HPL. Prior to the comparative analysis of HPL and L-HPL, commercially available HPL (PAN Biotech) was compared to seven batches of manufactured HPL to confirm sufficient product quality of our manufacturing protocol (Supplementary Figure S1C). Quality control measurements for pH and osmolality were performed using a pH-meter (CG 840, Schott) and an osmometer (OM815, Vogel), respectively. Total protein content was assessed using the modified biuret method on a Dimension Vista System (Siemens Healthineers). Potentiometric determination was used to measure potassium, sodium and chloride concentration on the Dimension Vista System (Siemens Healthineers).

#### Multiplex analysis of soluble mediators

Growth factor and cytokine concentrations were quantified using a customized LegendPlex assay (BioLegend) according to manufacturer’s instructions. The following soluble factors were analyzed: transforming growth factor-beta 1 (TGF-β1), vascular endothelial growth factor (VEGF), fibroblast growth factor-2 (FGF-2), endothelial growth factor (EGF), CC-chemokine ligand 5 (CCL5), intercellular adhesion molecule-1 (ICAM-1), platelet-derived growth factor-BB (PDGF-BB), platelet-derived growth factor-AA (PDGF-AA), brain-derived neurotrophic factor (BDNF), C-X-C motif ligand 1 (CXCL1), and vascular cell adhesion molecule-1 (VCAM-1).

#### Quantification of proliferation capacity, viability and hMSC phenotype

Proliferation capacity and cell viability were assessed in cell culture passage three at day 0 (24 h after seeding), day 4 and day 7 after seeding in 48-well tissue culture plates at a density of 1.8 × 10^3^ cells per well in 200 μL EM containing either 7% (v/v) HPL or 7% (v/v) L-HPL. Proliferation capacity was quantified using the CyQuant assay and cell viability was assessed using PrestoBlue cell viability reagent (both ThermoFisher Scientific) according to manufacturer’s instructions. Fluorescence quantification for both readouts was performed on a TECAN Infinite M200 Pro plate reader (Tecan Trading AG). Population doublings at day 4 and 7 were calculated according to the CyQuant fluorescence values using the following formulas: *log(day 4/day 0)/log(2)* and *log(day 7/day 0)/log(2)*, respectively. Phenotypic characterization of hMSC was performed at P3 after culture in HPL- or L-HPL-containing medium using flow cytometry. Following ISCT criteria, hMSCs were defined as CD14^−^ CD19^−^ CD34^−^ CD45^−^ CD73^+^ CD90^+^ CD105^+^ (Supplementary Figure S1B). The following anti-human antibody clones were used: CD14-APC-Cy7 (QA18A22), CD19-APC-Cy7 (HIB19), CD34-APCCy7 (581), CD45-APCCy7 (2D1), CD73-BV421 (AD2), CD90-PE (5E10), CD105-PerCP/Cyanine5.5 (43A3) (all BioLegend).

#### Cell differentiation

Osteogenic, adipogenic and chondrogenic differentiation of hMSCs were performed as described previously (Handke et al., 2023; Andrzejewska et al., 2019). Basal medium and media for osteogenic and adipogenic differentiation were supplemented with either 7% (v/v) HPL or 7% (v/v) reconstituted L-HPL. Briefly, cells from donor 1 were seeded at a density of 4,800 cells per well in 48-well tissue culture plates and cultured in EM for 24 h. After 24 h, osteogenic differentiation was induced by replacing EM with osteogenic medium (OM) containing low glucose DMEM, 10 mM β-glycerolphosphate disodium salt, 50 μML-ascorbic acid 2-phosphate sesquimagnesium salt, 100 nM dexamethasone (all Sigma-Aldrich), 100 U/mL penicillin, 100 μg/mL streptomycin and 2 mM L-alanyl-L-glutamine. Medium was changed twice a week and osteogenic differentiation potential was assessed by quantifying alkaline phosphatase (ALP) activity by measuring para-Nitrophenylphosphate (*p*NPP, Sigma-Aldrich) consumption after four and 7 days and by mineral matrix mineral quantification using alizarin red staining and subsequent colorimetric measurements on the TECAN plate reader after 18 days as previously described (Handke et al., 2023).

For adipogenic differentiation, cells from donor 1 were seeded at a density of 9,000 cells per well in 48-well tissue culture plates and EM was changed to adipogenic medium (AM) containing high glucose (4,500 mg/L) DMEM (PAN Biotech), 100 U/mL penicillin, 100 μg/mL streptomycin, 2 mM L-alanyl-L-glutamine, 1 μM dexamethasone, 2 μM insulin from bovine pancreas (Th. Geyer), 500 μM 3-isobutyl-1-methylxanthine (Sigma-Aldrich) and 100 μM indomethacin (Sigma-Aldrich) after 24 h of culture. Medium was changed twice a week. At culture day 14, cells were stained with 1 μg/mL 4′,6-Diamidin-2-phenylindol (DAPI) in dH_2_O and 0.1% Nile Red in phosphate-buffered saline (Bio&Sell GmbH). Fluorescence imaging of DAPI-stained cell nuclei and Nile Red-stained fat droplets was performed on a fluorescence microscope (Invitrogen EVOS FL). Fluorescence intensities were quantified using the TECAN plate reader. Nile Red signal was normalized to the corresponding DAPI signal to account for variations in total cell number.

For chondrogenic differentiation, cell spheroids were generated from 3 × 10^5^ cells of donor 2 by centrifugation at 400 × g in 15 mL conical tubes. The spheroids were cultured in chondrogenic medium containing high glucose (4,500 mg/L) DMEM, 100 U/mL penicillin, 100 μg/mL streptomycin and 2 mM L-alanyl-L-glutamine, 173 μML-ascorbic acid 2-phosphate sesquimagnesium salt, 0.1 μM dexamethasone, 0.35 mM proline (Sigma-Aldrich), 1 mM sodium pyruvate (PAN Biotech), 1.25 mg/mL human serum albumin (HSA; Biotest AG), 6.25 μg/mL insulin–transferrin–sodium selenite media supplement (Sigma-Aldrich) and 19.1 μM linoleic acid (Sigma-Aldrich). To induce chondrogenic differentiation, 10 ng/mL recombinant human TGF-β1 (BioLegend) was added and spheroids were maintained for 21 days. Medium was changed twice a week. Total protein content of spheroids was quantified using Pierce Coomassie Protein Assay Kit (ThermoFisher Scientific) according to the manufacturer’s instructions and proteoglycan content was quantified using a dimethylmethylene blue (DMMB) assay as described previously (Andrzejewska et al., 2019; Davis et al., 2011). For histological evaluation, the spheroids were fixed in 4% (v/v) formaldehyde (Carl Roth) for 1 h and stored in PBS until embedding in paraffin. Histological sections of the spheroids (7 µm) were stained with 1% alcian blue 8GX (Morphisto) and counterstained with nuclear fast red (Carl Roth). Spheroid size was assessed using area quantification in ImageJ of cross-sections based on light microscopy images taken after spheroid fixation.

### Descriptive and exploratory statistics

GraphPad PRISM 8.4.3 was used to display all data and for statistical comparison of the two groups. The determination of parametric or non-parametric tests and plotting of mean values with standard deviation (SD) or median values with interquartile range (IQR) was based on the Shapiro-Wilk normality test. Samples sizes, i.e., all individual values, are shown in the corresponding graphs. The groups were not blinded during the analyses and statistical tests. For all analysis of corresponding HPL and L-HPL batches, paired t-test was applied. Further details on the statistical tests applied are indicated in the figure captions.

## Results

### Parameters of routine quality control

**FIGURE 2:**
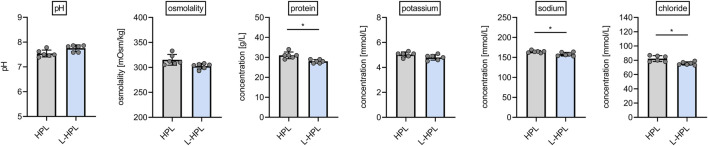
Routine quality control measurements of HPL and reconstituted L-HPL. Six corresponding batches of HPL and reconstituted L-HPL were analyzed regarding pH, osmolality, total protein, potassium, sodium and chloride content [mean ± SD; paired t-test; *, p < 0.05].

Aiming to test whether lyophilization presents a suitable option for improved storage and stability of HPL, we generated six batches of HPL and L-HPL from four pooled PCs (containing platelet concentrates from 16 individual donors) according to the manufacturing process detailed in Figure 1. All six batches of HPL and L-HPL were then subjected to previously suggested standard platelet lysate quality control measurements (De Korte et al., 2024), including pH, osmolality and total protein content as well as potassium, sodium and chloride content (Figure 2).

While pH, osmolality and potassium content were not altered between HPL and reconstituted L-HPL batches, a slight but significant reduction (p = 0.014) in total protein content of reconstituted L-HPL was observed [g/L, mean ± SD]: HPL, 31.0 ± 1.7; L-HPL, 28.0 ± 0.9. In addition, sodium and chloride contents were significantly reduced (sodium, p = 0.045; chloride, p = 0.020) in reconstituted L-HPL [mmol/L, mean ± SD]: chloride HPL, 82.3 ± 4.1; chloride L-HPL, 75.5 ± 2.3; sodium HPL, 164.3 ± 2.8; sodium L-HPL, 158.0 ± 4.6. Taken together, total protein, chloride and sodium contents were slightly reduced in reconstituted L-HPL batches compared to the corresponding HPL batches.

### Quantification of soluble factors

**FIGURE 3:**
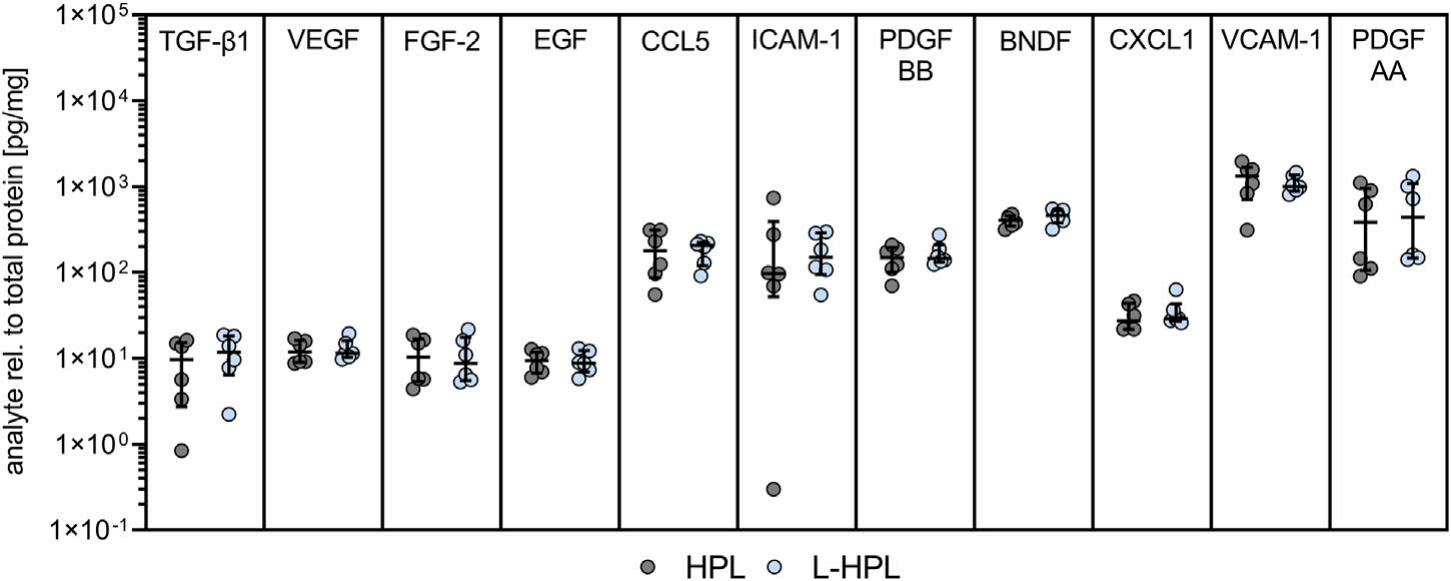
Growth factor and cytokine concentrations in HPL and L-HPL. Levels of indicated molecules were normalized to total protein content for each corresponding batch of HPL and L-HPL [n = 6, median with IQR, paired t-test].

Another important quality control parameter for HPL is the concentration of relevant platelet-related growth factors and cytokines, including TGF-β1, PDGF-AA and PDGF-BB (De Korte et al., 2024). To test whether lyophilization had an impact on growth factor and cytokine levels, a range of 11 soluble mediators were compared between HPL and L-HPL batches (Figure 3).

For all of the tested soluble factors, no significant differences were detected between HPL and reconstituted L-HPL, indicating that the added freeze-drying step does not lead to a loss of important mediators of cell growth and functionality.

### Yield, proliferation and viability of mesenchymal stromal cells

To directly test whether lyophilization affects HPL functionality as cell culture supplement, we compared cell yield, proliferation capacity and metabolic activity as an indicator for cell viability of hMSCs from the bone marrow in EM containing either HPL or L-HPL.

**FIGURE 4:**
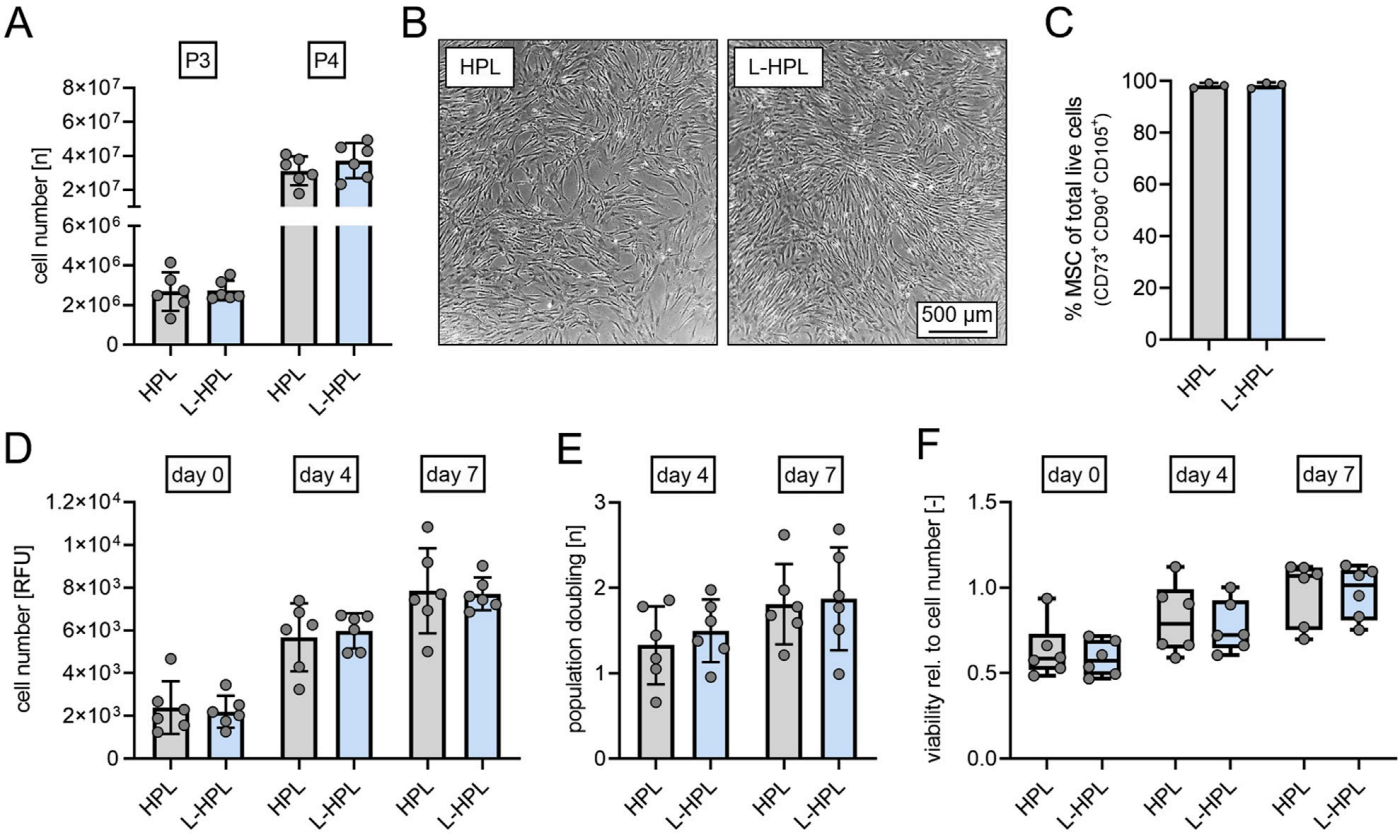
Cell yield, proliferation capacity and viability of human mesenchymal stromal cells cultured in medium containing either HPL or L-HPL. **(A)** Cell numbers quantified at cell culture passage 3 (P3) and passage 4 (P4). **(B)** Representative phase contrast imaging of cells cultured in EM containing either HPL or L-HPL at P3. **(C)** Percentage of MSC at cell culture passage P3 determined by flow cytometry. MSC identity was defined according to ISCT criteria as CD14^−^CD19^−^CD34^−^CD45^−^CD73^+^CD90^+^CD105^+^. **(D)** Relative cell numbers determined by DNA quantification at 24 h (day 0), day 4 and day 7 after seeding. **(E)** Proliferation rates at day 4 and day 7 after seeding. **(F)** Metabolic activity normalized to cell number as a marker for cell viability at 24 h (day 0), day 4 and day 7 after seeding [normally distributed data: paired t-test, mean ± SD; non-normally distributed data: Wilcoxon matched-pairs signed rank test, Boxplots, whiskers (min–max)].

At cell culture passages 3 and 4, total cell numbers of hMSCs did not differ between cultures containing HPL and L-HPL (Figure 4A). Routine phase contrast microscopy of expanded cells did not indicate any obvious morphological differences between the two groups (Figure 4B). Phenotypic characterization at cell culture passage 3 following ISCT criteria (Dominici et al., 2006) confirmed hMSC identity with >98% of cells being CD14^−^ CD19^−^ CD34^−^ CD45^−^ CD73^+^ CD90^+^ CD105^+^ in both cell cultures containing HPL and L-HPL (Figure 4C; Supplementary Figure S1B). Cell number determination by DNA quantification 24 h after seeding (day 0) and at days 4 and 7 and calculation of the population doublings at days 4 and 7 did not indicate any difference regarding proliferation of hMSCs cultured in media containing either HPL or L-HPL (Figures 4D, E). Furthermore, similar levels of metabolic activity were detected in cells cultured with HPL- and L-HPL-containing medium (Figure 4F). Together, these findings demonstrate that L-HPL is equally suitable for expansion of hMSCs and that the lyophilization process does not negatively affect the growth-supporting features of HPL.

### Trilineage differentiation potential of mesenchymal stromal cells

Successful support of hMSC cultures does not only comprise their ability to proliferate, but also to maintain their potential to differentiate into adipogenic, osteogenic and chondrogenic lineages. In order to test whether L-HPL can support the trilineage differentiation potential of hMSCs as well as HPL, we compared the differentiation of hMSCs induced by the respective adipogenic and osteogenic media supplemented with either HPL or L-HPL. Since chondrogenic media does not contain blood-based additives, hMSCs differentiated to the chondrogenic lineage were expanded in EM supplemented with either HPL or L-HPL for one additional cell culture passage.

**FIGURE 5:**
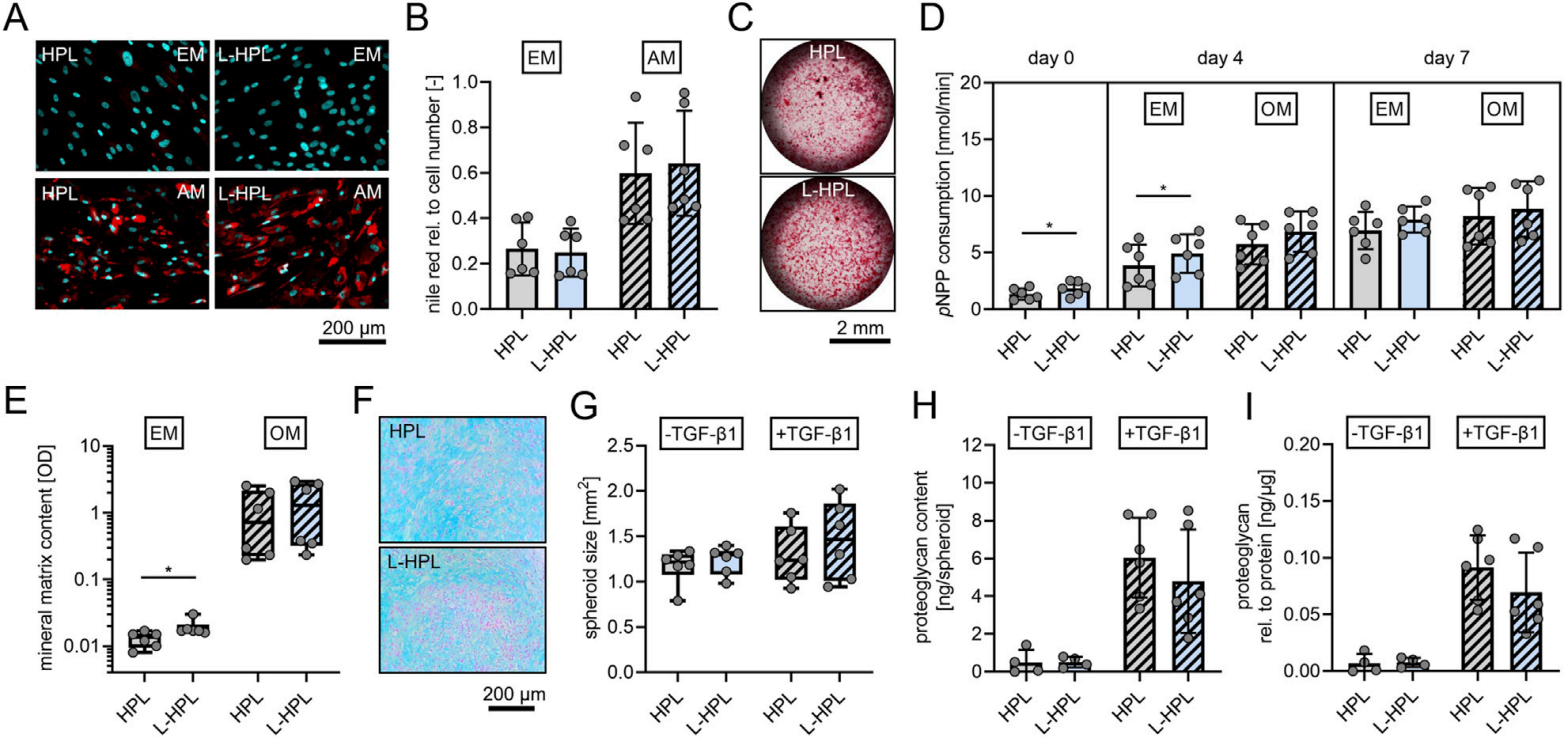
Trilineage differentiation potential of mesenchymal stromal cells cultured in expansion medium containing either HPL or L-HPL. **(A)** Representative fluorescence images of hMSCs after staining with nile red (red, lipid droplets) and DAPI (cyan, nuclei) at day 14 of culture in expansion medium (EM) or adipogenic medium (AM) containing either HPL or L-HPL. **(B)** Corresponding quantification of nile red signals relative to DAPI signals of cells cultured in EM or AM containing either HPL or L-HPL. **(C)** Representative bright field microscopy images of hMSCs after staining with alizarin red (red, calcium deposits) at day 18 of culture in OM containing HPL or L-HPL. **(D)** Quantification of *p*NPP consumption indicating alkaline phosphatase activity at day 0 (24 h after seeding), day 4 and day 7 of culture in EM or osteogenic medium (OM) containing either HPL or L-HPL. **(E)** Quantification of the mineral matrix content following alizarin red staining at day 18 of culture in EM or OM containing either HPL or L-HPL. **(F)** Representative images of alcian blue stained chondrogenic spheroid sections at day 21 of 3D culture of hMSCs expanded in either HPL or L-HPL. **(G–I)** Quantification of **(G)** areas of spheroids’ cross sections, **(H)** total proteoglycan content of spheroids at day 21 of 3D culture without (-TGF-β1) and with chondrogenic stimulus (+TGF-β1), and **(I)** total proteoglycan content of spheroids normalized to the total protein content of spheroids. [normally distributed data: paired t-test, mean ± SD; non-normally distributed data: Wilcoxon matched-pairs signed rank test, Boxplots, whiskers (min–max); *p < 0.05].

Adipogenic differentiation was confirmed through Nile Red staining of lipid droplets (Figure 5A). Quantification of fluorescence intensities did not indicate any significant differences between the two groups in terms of adipogenic differentiation potential of non-stimulated or stimulated cells (Figure 5B). Osteogenic differentiation was confirmed through alizarin red staining of mineralized matrix (Figure 5C). As an early marker for osteogenic differentiation, ALP activity was quantified. At day 0 (24 h after seeding), cells cultured in L-HPL showed a significantly higher cellular ALP activity (p = 0.030) compared to cells cultured in HPL [nmol/min, mean ± SD]: HPL, 1.35 ± 0.51; L-HPL, 1.86 ± 0.62. At day 4, non-stimulated cells also showed a significantly higher cellular ALP activity (p = 0.040) compared to cells cultured in HPL [nmol/min, mean ± SD]: HPL, 3.88 ± 1.83; L-HPL, 4.92 ± 1.69. At day 4, cells stimulated with osteogenic medium did not show significantly higher cellular ALP activity compared to cells cultured in HPL and at day 7, quantification of ALP activity did not indicate any significant differences in terms of osteogenic potential between the two groups of non-stimulated or stimulated cells (Figure 5D). Colorimetric quantification of the mineral matrix content at cell culture day 18 did not indicate any significant differences in terms of osteogenic potential between the two groups following stimulation with osteogenic medium. Non-stimulated cells cultured in osteogenic medium containing L-HPL mineralized significantly more matrix compared to those cells culture in HPL [OD, median with (IQR)]: HPL, 0.0135 (0.0060); L-HPL, 0.0170 (0.0043). Cells of both groups stimulated with osteogenic medium did not differ significantly in terms of their potential for matrix mineralization (Figure 5E). Thus, the minor increase in ALP activity and matrix mineralization in cells cultured with L-HPL-containing expansion medium are most likely not biologically relevant, as those differences were not maintained after culture in osteogenic medium. Overall, HPL and L-HPL containing media supported osteogenic differentiation of hMSCs equally well.

Chondrogenic differentiation was confirmed through alcian blue staining, indicating glycosaminoglycans of chondrogenic spheroids sections (Figure 5F). Quantification of the area of chondrogenic spheroid cross sections (Figure 5G), protein quantification of the spheroids (data not shown), quantification of total proteoglycan content (Figure 5H), and normalized proteoglycan to protein content of the spheroids (Figure 5I) did not indicate any differences between the two groups regarding the cells’ potential for chondrogenic differentiation with or without stimulation of chondrogenic differentiation with TGF-β1.

In summary, these findings show that L-HPL supports hMSC culture and differentiation equally well as HPL, thus demonstrating that lyophilization of HPL preserves its essential features as cell culture supplement.

## Discussion

The paramount aim of this study was to establish a protocol for L-HPL manufacturing allowing for production under good manufacturing practice conditions, and to rigorously test L-HPL compared to conventional HPL regarding relevant quality controls and functional parameters in hMSCs culture. We could show that lyophilization as a whole does not impact HPL quality and maintains all features of conventional HPL in supporting the expansion and differentiation of hMSCs *in vitro*. Thus, we conclude that lyophilization offers a valuable sustainable and energy-efficient option to render HPL more stable, easier to handle and better to apply as cell culture supplement while maintaining all essential quality features.

In light of rising interest in cell-based therapies and regenerative approaches in different fields of medicine, the need for non-xenogenic alternatives to FCS in human cell cultures is increasing (Burnouf et al., 2023). The benefits of using platelet lysates to provide growth factors and nutrients for *in vitro* cell expansion have been described for various cell types, including primary hMSCs from different tissues. However, the widespread use of HPL is hampered by its limited storage stability, precipitations and batch variations. In our hands, a pool of platelet material from 16 donors, as suggested sufficient for HPL batch stability previously (Agostini et al., 2017), still contained certain variations in growth factor concentration. Thus, expanding the donor pool to 20–24 for improved batch stability should be considered as an option for future applications of HPL.

One possibility to improve the usability of HPL is to freeze-dry the solution, thus providing a stable product that can be stored at 4°C or even RT and reconstituted when needed. In a recent study, MSCs isolated from umbilical cord tissue (UCT) have been successfully expanded and differentiated *in vitro* using lyophilized HPL (Marino et al., 2023), while a previous study has successfully used L-HPL in an *in vivo* wound healing model (Notodihardjo et al., 2019). The latter compared different storage conditions of small aliquots of HPL, including cryopreservation and lyophilization, and found a significant loss of growth factors PDGF-BB, VEGF and TGF-β after lyophilization which the authors attribute to the loss of material during the freeze-drying process (Notodihardjo et al., 2019). Here, we expanded the systematic comparison of six corresponding larger batches of HPL and L-HPL to 11 growth factors and cytokines and did not observe alterations after lyophilization. Thus, establishing a freeze-drying process that warrants the collection of the complete dried material is critical, especially when working with small volumes.

To date, the use of L-HPL in in vitro cell cultures has been demonstrated for fibroblast cultures (Notodihardjo et al., 2019), UCT-derived MSCs (Marino et al., 2023) and in different *in vitro* wound healing assays using human keratinocytes (Buscemi et al., 2024) and human corneal epithelial cell lines (Chen et al., 2018). Although these studies showed promising results, a direct comparison of HPL and L-HPL regarding differentiation of MSCs was lacking. Our findings confirm that L-HPL supports the expansion and proliferation of bone marrow-derived hMSCs equally well as HPL and, importantly, maintains the identity and trilineage differentiation potential of these cells. Together, these initial studies on the use of L-HPL are the first important contributions to better describe and define this form of preservation of HPL. Based on these findings, future studies investigating the influence of L-HPL on other clinically relevant aspects of MSC biology, including their immunomodulatory properties, should help to define its potential for use in cell therapy.

With this novel approach to make HPL more stable to use, more possibilities of application are conceivable that go beyond use in biomedical research. Platelet derived products such as platelet-rich plasma (PRP) are already being used in the clinic for regenerative approaches in orthopedic surgeries and for musculoskeletal diseases, to support healing and improve tissue regeneration (Sánchez et al., 2022). Currently, PRP is produced from autologous plasma of the patient during surgery, a method that is both time-consuming and significantly influences the therapeutic effect due to varying quality of the PRP (Niemann et al., 2023). A standardized platelet product that can be readily used during the procedure would help to improve reliability and outcome. Being pre-produced from platelet material of multiple healthy donors, L-HPL has superior lot-to-lot consistency and could provide a stable off-the-shelf option with better clinical applicability, alone or in combination with biomaterial-based regenerative approaches. While future developments in terms of controlled production and a regulatory framework with regard to the use in cell therapy manufacturing are still required to take L-HPL availability to the next level, we believe that our evaluation of L-HPL manufacturing and quality presents an important contribution to the field of platelet derivatives.

## Data Availability

The raw data supporting the conclusions of this article will be made available by the authors, without undue reservation.
