# Supplementary material for: Lyophilized human platelet lysate: manufacturing, quality control, and application
Source: Front Cell Dev Biol. 2025 Jan 27;13:1513444. doi: 10.3389/fcell.2025.1513444 (PMC11807961; doi:10.3389/fcell.2025.1513444)
Supplement: Supplementary file 1 [file DataSheet1.pdf]

A

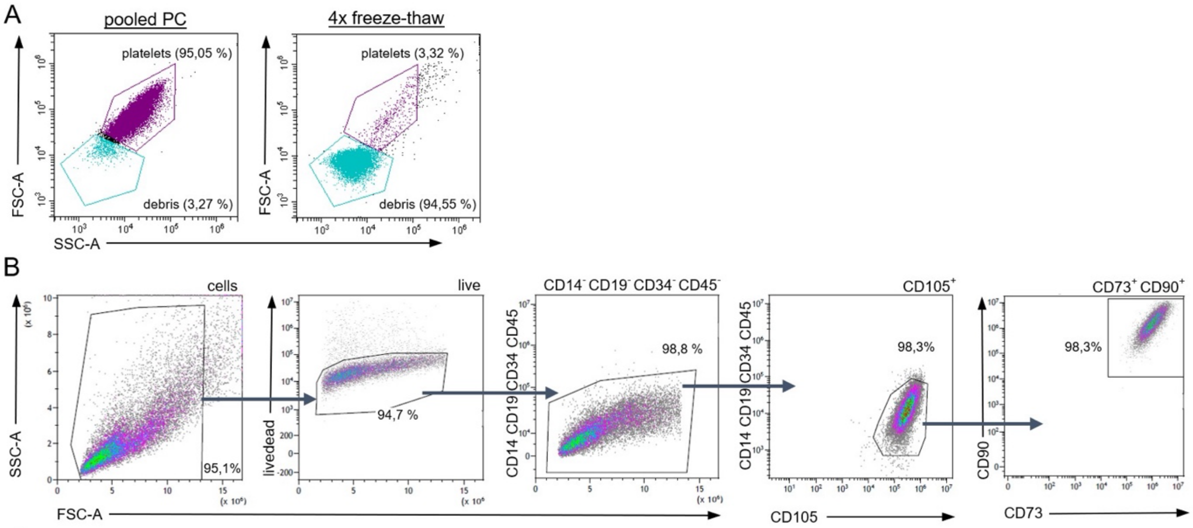

C

	PAN HPL	HPL (n=7)
Na [mmol/L]	152	174 ± 8,6
K [mmol/L]	4,8	5,4 ± 0,32
Cl [mmol/L]	86	87 ± 5,1
pH	7,6	7,5 ± 0,06
Osmolality [mOsm/kg]	291	280 ± 14,1
Protein [g/L]	19	26 ± 3,6

Supplemental Figure 1.

(**A**) Representative flow cytometry gating strategy to identify intact platelets and debris in the original pooled platelet concentrate (PC) and after four cycles of freeze-thaw. Plots show events following pre-gating for CD61$^+$ cells.

(**B**) Representative flow cytometry gating strategy to identify MSC phenotype according to ISCT criteria.

(**C**) Quality control parameters of commercially available HPL from PAN Biotech and seven batches of manufactured HPL.
